# Examining rater and occasion influences in observational assessments obtained from within the clinical environment

**DOI:** 10.3402/meo.v21.29279

**Published:** 2016-02-23

**Authors:** Clarence D. Kreiter, Adam B. Wilson, Aloysius J. Humbert, Patricia A. Wade

**Affiliations:** 1 Department of Family Medicine, University of Iowa College of Medicine, Iowa City, IA, USA; 2 Office of Consultation and Research in Medical Education, University of Iowa College of Medicine, Iowa City, IA, USA; 3 Department of Surgery, Indiana University School of Medicine, Indianapolis, IN, USA; 4 Office of Undergraduate Medical Education, Indiana University School of Medicine, Indianapolis, IN, USA; 5 Office for Mentoring and Student Development, Medical Student Affairs, Indiana University School of Medicine, Indianapolis, IN, USA

**Keywords:** clinical ratings, generalizability theory, clinical skills

## Abstract

**Background:**

When ratings of student performance within the clerkship consist of a variable number of ratings per clinical teacher (rater), an important measurement question arises regarding how to combine such ratings to accurately summarize performance. As previous G studies have not estimated the independent influence of occasion and rater facets in observational ratings within the clinic, this study was designed to provide estimates of these two sources of error.

**Method:**

During 2 years of an emergency medicine clerkship at a large midwestern university, 592 students were evaluated an average of 15.9 times. Ratings were performed at the end of clinical shifts, and students often received multiple ratings from the same rater. A completely nested G study model (occasion: rater: person) was used to analyze sampled rating data.

**Results:**

The variance component (VC) related to occasion was small relative to the VC associated with rater. The D study clearly demonstrates that having a preceptor rate a student on multiple occasions does not substantially enhance the reliability of a clerkship performance summary score.

**Conclusions:**

Although further research is needed, it is clear that case-specific factors do not explain the low correlation between ratings and that having one or two raters repeatedly rate a student on different occasions/cases is unlikely to yield a reliable mean score. This research suggests that it may be more efficient to have a preceptor rate a student just once. However, when multiple ratings from a single preceptor are available for a student, it is recommended that a mean of the preceptor's ratings be used to calculate the student's overall mean performance score.

Academic assessment plans in clinical medical education commonly rely on naturalistic observations of performance within healthcare delivery settings. Because these observational assessments play such a prominent role in medical education, it is important that the scores they produce be as accurate and informative as possible. Ratings obtained within a real clinical environment are attractive because they are thought to reflect the higher dimensions of Miller's often cited pyramidal taxonomy of knowledge and skills (Shows How & Does) (1). Although there are substantial threats to validity when using these assessments (2, 3), empirical investigations have shown that when mean scores are based on multiple independent ratings, a mean score is capable of providing a reliable summary of learners’ performance within the clinical environment (3–8).

At most academic medical centers, medical students receive ratings from clinical teaching faculty who complete one or more standardized rating forms designed to quantify performance in highly unstandardized clinical/instructional interactions. In these learning situations, it is quite common for a learner to receive many ratings, each reflecting one of the multiple opportunities (occasions) that a teacher has to observe a learner. Because an occasion to provide these ratings depends upon the highly variable schedule of the teachers, patients, and learners, a mean summary score can be based upon a varying number of ratings from each clinical teacher involved in the student's clinical instruction. For example, depending on the number of opportunities (occasions) afforded to observe and interact with a particular learner (medical student), some teachers (preceptors) may rate a learner just once, whereas other teachers, having had more occasions to interact with a learner, might provide multiple ratings for a single student over the course of a clerkship. When students’ ratings consist of a variable number of ratings per clinical teacher (rater), an important measurement question arises regarding how best to combine these ratings to accurately summarize performance. Although the research literature does report on generalizability (G) studies of clinic-based ratings, these studies have not modeled occasion and rater facets in a way that allows for an informed judgment regarding the best method for summarizing multiple ratings from a single clinical teacher (3–8). Given the lack of empirical evidence regarding the independent influence of raters and occasions on score variance, clerkship directors are currently required to use their intuitive judgment regarding how to generate scores that best summarize performance across raters and occasions.

It is interesting to consider the measurement implications of the two simplest methods for calculating a mean performance score for students with a variable number of ratings from each rater. The easiest method, to ignore the identity of the rater and simply average across all rating occasions, is likely to assign too large a weight to raters who have submitted the most observations for a learner. This can be especially problematic for observational ratings collected within clinical settings where systematic and interaction rater effects are thought to be quite pronounced (2, 3). To correct for this, clerkship directors could average across a single rater's observations for each learner and use this mean rating to calculate a learner's average score across raters. Although this assigns approximately equal weight to each rater, the mean rating will not reflect the number of observational opportunities. In which case, a rater who has had extensive opportunity to observe and rate a learner will be assigned the same weight in the summary score as a rater who has provided just one rating and has had comparatively less contact with the learner. The reliability of the summary scores from these two calculation methods can be very different depending on the number of occasions and raters. The current research literature does not offer guidance on how to optimally combine ratings to maximize the reliability of a summary score. Generalizability (G) theory is useful in answering such questions because, unlike classical test theory, G theory allows researchers to estimate the influence of each error source as a variance component (VC). Therefore, with an appropriately designed study, this method is capable of estimating the individual influence of rater and occasion.

Previous G studies of observational assessments within the clinical environment have shown that when ratings are collected across multiple occasions, each with a single unique rater, a reasonably reliable mean score for summarizing a learner's overall performance can be obtained with 8–12 independent ratings (3–8). Although these studies provide insight into how reliable mean scores are likely to be given various numbers of independent ratings, they do not estimate the level of information provided by multiple sequential ratings by the same rater. Rather, previous G studies have confounded the effects of rating occasion and rater in their analysis. That is, the G study calculations were performed on data samples that included a different rater on each rating occasion for each individual student being evaluated. With the data structured in this fashion, G studies cannot estimate the reliability of scores generated from multiple rating occasions by the same rater. This highlights a practically important gap in our knowledge of rating situations in which learners are rated frequently (e.g., after each daily clinical shift) by the same rater.

Estimating occasion effects distinct from rater effects is important in any assessment protocol that uses a small number of raters to perform multiple ratings (9–11). This research examines ratings collected across multiple raters and occasions. It addresses the important question of how to interpret mean ratings that are calculated across a varying number of raters and occasions. Although the answer to this question is largely concerned with reliability, it also yields important validity evidence. Understanding the relative influence of the various sources of error provides fundamental information about how the rating process interacts with student performance.

## Methods

### Data

During the 2012–2013 and 2013–2014 academic years, the geographically diverse Indiana University School of Medicine evaluated 592 third-year medical students multiple times during their clerkship rotation in emergency medicine (EM). Preceptors rated their students with a standardized clinical evaluation form composed of seven items that each utilized a 5-point Likert-type rating scale (1=unacceptable/5=exceptional). Preceptors could elect to omit an item if they felt they had insufficient information to provide a rating. Over the course of the 2 years, the 592 students were collectively evaluated a total of 9,423 times. In all, 411 raters, made up of EM faculty, EM volunteer faculty, and EM residents were involved in the clinical teaching and assessment of these learners across 26 hospital sites within the state of Indiana. Ratings were typically completed at the end of a shift, and students received multiple ratings during the clerkship. With this assessment design, students were often rated multiple times by the same rater. Table 1 displays a summary of the rating data used. The use of this rating data was approved by the institutional review board.

**Table 1:** Summary of rating data

N of students (Total no. ratings)	Total no. of raters	Mean rating	Score range	SD of score	Average no. of ratings (raters) per student
592 (9,423)	411	4.02	1.5–5.0	0.646	15.9 (7.6)

### The design/analysis

The primary challenge in constructing a model for estimating VCs from observational ratings obtained in a clinical setting involves conceptual issues related to the nesting of the occasion and rater facets. Considering the objectives of this research, it would be ideal to conduct a G study capable of estimating all VCs related to the rater and occasion facets and their interactions. Unfortunately, the structure of the data collected in the rating context of this study restricts what VCs can be estimated. In practice, the occasion to rate is nested within a rater, and raters are functionally nested within students. The G study design describing the rating data in this study is an occasion-nested-within-rater-nested-within-person (o:r:p) model. Consistent with the notation used by Brennan and Kreiter (12, 13), ‘p’ represents the ‘person’ facet (learners as the ‘object-of-measurement’); ‘r’ represents the ‘rater’ facet; and ‘o’ represents the ‘occasion’ facet. The ‘:’ designates that the facets on the left of the symbol are nested within the facets on the right of the symbol. Table 2 describes which VCs from the fully crossed model (p x r x o) are incorporated into the three VCs that are obtained from the fully nested model. The G coefficient estimate of reliability from the o:r:p model is calculated as shown in Equation 1.

$$\rho^{2}=\sigma^{2}(p)/(\sigma^{2}(p)+[\sigma^{2}(r:p)/n_{r}]+[\sigma^{2}(o:r:p)/n_{r}*n_{o}])$$

**Table 2:** Variance components from the (o:r:p) model mapped to the (p×r×o) model

Variance components from the fully nested model – (o:r:p)	Variance components from the fully crossed model – (p×r×o) that are included in each VC estimate
p	p
r:p	r, pr
o:r:p	o, po, ro, pro(e)

**Equation 1:** Generalizability coefficient

The ratings used in the G study were selected using stratified random sampling from those students who had been rated two or more times by three or more raters. The stratified sampling allowed the use of balanced nested data. The final G study dataset contained three unique raters for each student with each of the three raters rating the student on two different occasions. Decision (D) studies estimated the reliability given various numbers of raters and occasions. The estimated G coefficients from the D studies were compared with coefficient alphas that were calculated with multiple random balanced samples of varying numbers of raters and occasions nested with raters. The samples used to compute the coefficient alphas contained various numbers of raters who each rated the student multiple times.

## Results

To maximize the sample sizes for a balanced G study design, ratings for students who had been rated by three or more raters who had each rated the student on two or more occasions were eligible for inclusion in the analysis. In total, 299 students who each experienced three unique raters rating the student on two occasions (1,794 occasions) were included in the G study. An SAS^®^ random numbering program was used for sampling.

Table 3 displays the results of the G study analysis. The variance related to person accounted for about one-fourth (23%) of the variance and the error associated with raters accounted for well over half (59%) of the observed variance. The VC related to occasion (o:r:p) accounted for just 18% of the total variance. The small magnitude of the standard errors (SEs) relative to the VC measures (0.04–0.16 in size) suggests the VCs were estimated with precision.

**Table 3:** G study table for the o:r:p design

Facet	DF	VC	%	SE
p	298	0.0996490	23	0.0164167
r:p	598	0.2346347	59	0.0158407
o:r:p	897	0.0759490	18	0.0035832

**Fig. 1:**
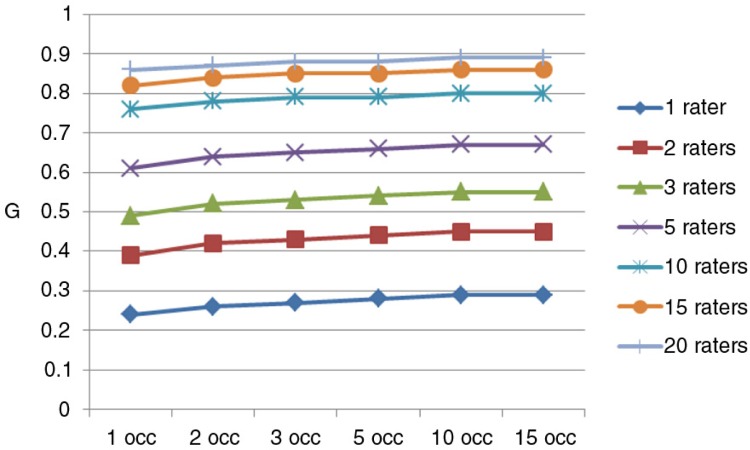
D Study – Generalizability as a function of number of raters and occasions.

Table 4 displays estimated G coefficients for 1–20 raters and 1–15 nested occasions. This table indicates that having a rater evaluate a student on multiple occasions does little to enhance the reliability of student performance scores. On the other hand, increasing the number of raters makes a dramatic impact on the reliability of the mean score. This result is graphically depicted in Fig. 1.

**Table 4:** D study table – G coefficients as a function of number of raters and occasions

	Number of raters						
	
Occasion nested within rater	1	2	3	5	10	15	20
1	0.24	0.39	0.49	0.61	0.76	0.82	0.86
2	0.26	0.42	0.52	0.64	0.78	0.84	0.87
3	0.27	0.43	0.53	0.65	0.79	0.85	0.88
5	0.28	0.44	0.54	0.66	0.79	0.85	0.88
10	0.29	0.45	0.55	0.67	0.80	0.86	0.89
15	0.29	0.45	0.55	0.67	0.80	0.86	0.89

Table 5 displays the results of taking the mean of two ratings by the same rater versus using just one random rating to calculate a summary score. As predicted in the D study, in actual practice, a mean score composed of two ratings per rater is approximately as reliable as using a single random rating from each rater.

**Table 5:** Alpha coefficients for random samples from 41 to 423 students that where rated more than once by 2–6 raters

No. of times student rated by the same rater 2 or more times	No. of students	Alpha coefficients for two random ratings from each of 2–6 random raters (4 random samples per group) (Alpha coefficients for 2–6 raters rating each student once)
1	510	–
2	423	0.43 (0.37, 0.39) 0.34 (0.27, 0.33) 0.35 (0.36, 0.32) 0.43 (0.39, 0.42)
3	299	0.52 (0.51, 0.47) 0.49 (0.48, 0.44) 0.53 (0.49, 0.49) 0.52 (0.48, 0.50)
4	190	0.56 (0.52, 0.53) 0.52 (0.46, 0.52) 0.57 (0.54, 0.52) 0.53 (0.50, 0.50)
5	92	0.63 (0.58, 0.61) 0.62 (0.60, 0.59) 0.64 (0.60, 0.62) 0.56 (0.49, 0.58)
6	41	0.68 (0.71, 0.60) 0.70 (0.69, 0.68) 0.68 (0.64, 0.68) 0.69 (0.66, 0.67)

## Discussion

The results provide reliability and validity evidence regarding the performance of clinic-based ratings. Clearly, the particular patient with which the students interact is not an influential determinate of the rating received. The common interpretation of ‘case specificity’ as an explanation for the low correlation between performances does not apply to these ratings (2). As Table 2 implies, the actual influence of the combined occasion and person-by-occasion variances is certainly less than 18% of the total variance as the o:r:p VC also contains other sources of error variance and unmodeled error (e) that is confounded with the highest order factor (o:r:p). As clinical cases are a hidden facet nested within occasion, these results suggest a modest impact related to ‘case-specific’ factors.

Having a single rater repeatedly rate a learner on different cases is unlikely to generate a reliable score. The scores awarded are primarily dependent on the rater assigned to the student. Unfortunately, this study could not independently estimate the proportion of error attributable to systematic rater effects (r) (stringency vs. leniency) versus the rater-by-person interaction (pr) (12). As indicated in Table 2, the nested nature of the data results in a single r:p VC estimate that represents the confounded systematic and interaction rater effects as the sum of the r and rp VCs from the fully crossed model. The validity implications of systematic and interaction rater error are different and it seems logical that rater-by-person interaction error variance might have a more negative implication regarding validity compared with systematic rater error. Although both are modeled as random error and can be reduced by increasing the number of raters, the implications of the two sources of error are different. The systematic error is by definition random and can be accurately estimated and easily interpreted as resulting from differences in rater stringency; on the other hand, the interaction effect (rp) might imply that raters are assessing different aspects of student performance (or a student characteristic) and that there is disagreement regarding what the ratings should reflect. From a validity standpoint, the systematic effect is likely less of a threat as it implies only that the raters have different expectations regarding the level of performance expected, but not that there is disagreement about what should be assessed. Conversely, if most of the r:p error variance is attributable primarily to the person-rater (pr) interaction, this could imply the presence of construct-irrelevant variance related to extraneous personality factors or ill-defined measurement objectives. Furthermore, unlike systematic rater effects, for the pr interaction there is no guarantee that the origins of this error are entirely random. For example, suppose that construct-irrelevant personality dimensions are impacting ratings in a consistent direction (+/−), but the magnitude is inconsistent across raters. This would show up partially as a person-by-rater interaction effect (pr), but part of this error would also be incorporated as ‘true’ score variance (p). Only additional research using a fully crossed model can address this question.

These results have strong implications for designing as assessment strategy. In particular, it is not particularly useful to have the same rater periodically evaluate a student on different cases during the clerkship. The overall efficiency of the rating process may be enhanced by asking the preceptor to rate the student just once at the end of the student–preceptor interaction. Also, when multiple ratings per preceptor are available for a single student, it is recommended that course directors use a single rater's mean across occasions (cases/forms) to generate a student's mean score.
